# Making case‐based learning work and why context matters

**DOI:** 10.1111/medu.70205

**Published:** 2026-03-11

**Authors:** Skye Nandi Adams

**Affiliations:** ^1^ Department of Speech Pathology and Audiology, School of Human and Community Development, Faculty of Humanities University of the Witwatersrand Johannesburg South Africa

## Abstract

#CBL isn't one‐size‐fits‐all. Context shapes the mechanisms that drive learning. This commentary applies a realist lens to show how case design, facilitation & resources determine what works, for whom. #MedEd

Case‐based learning (CBL) is widely used in the education and training of healthcare students internationally and is recognised for its potential to promote clinical reasoning, learner engagement and the integration of theory and practice.^1^, ^2^ However, effective implementation of CBL requires considerable investment in time, resources and faculty development. While the benefits of CBL are well established, far less attention has been paid to how this pedagogical approach is implemented and experienced in low‐ and middle‐income countries (LMICs) and other resource‐constrained settings.

Recent work by Daly et al^3^ highlights several mechanisms underpinning CBL that have been under‐theorised in previous research, including the central role of peer dialogue in facilitating learning. Importantly, this review also foregrounds the significance of *context* in shaping how CBL is experienced and whether intended learning outcomes are achieved.^3^ While the review identifies key mechanisms underpinning effective CBL, I argue that these mechanisms are inseparable from the institutional, socio‐economic and clinical environments in which learning occurs. Students trained in LMIC often experience contextual complexities that are overlooked when cases are directly transposed from high‐income settings. Developing cases that reflect students' own clinical environments, patient populations and health system constraints is essential to ensure relevance and engagement.^4^ Authentic cases grounded in real‐world practice have been shown not only to enhance learning outcomes but also to improve student confidence, motivation and satisfaction.^5^ Although context is critical, the development of CBL in resource‐constrained settings presents several challenges. Challenges include the time and expertise required to develop contextually appropriate cases, limited opportunities for facilitator training and variable levels of student preparedness for inquiry‐based learning approaches such as CBL.

To mitigate these challenges, careful consideration must be given to how resources, time and training are allocated in the design and delivery of CBL. Emerging evidence suggests that it is not the technical sophistication or production quality of cases that drives learning, but rather their authenticity and alignment with learning outcomes.^4^, ^5^ As such, educators in resource‐constrained settings may benefit from prioritising short cases with progressive disclosure over lengthy narratives and from drawing on routinely available local clinical data. The use of structured frameworks for case development^4^ allows for the identification of a minimum dataset tailored to specific learning objectives while also supporting consistency in facilitation. Embedding prompts for reflection and decision‐making within cases can further promote critical thinking and deeper learning.^6^

Staff shortages and limited faculty capacity remain persistent challenges in many universities globally, but particularly in LMICs.^7^ One strategy to optimise available resources is the intentional use of postgraduate students as near‐peer facilitators.^8^ Near‐peer teaching involves students in the same programme facilitating a student at a lower level in their education and can expand facilitation capacity. Near‐peer teaching can provide relatable role models for undergraduate students and reduce the burden on academic staff. Furthermore, as noted by Daly et al,^3^ the use of near‐peer facilitators also provides students with a role model for approaching clinical problems and one that focuses more on guidance over only teaching and using a very didactic approach.

Collaboration is key when using CBL and navigating clinical uncertainty in a resource‐constrained setting. CBL requires collaboration not only among university‐based lecturers but also with clinical educators involved in student supervision. Training clinical educators to develop and facilitate cases using shared frameworks can support the creation of a reusable, contextually relevant case bank and enable the integration of interdisciplinary perspectives. Such collaborative approaches also provide a foundation for developing communities of practice, which offer sustainable spaces for sharing resources, reflecting on teaching experiences and collectively addressing implementation challenges.

More recently, there has been growing interest in simulation‐based learning within health professions education. CBL can be meaningfully integrated into simulation through strategies such as structured role‐play, virtual reality or the use of digital representations of clinical scenarios. Role‐play and virtual reality have both shown to increase students' critical thinking, engagement and academic outcomes across various health disciplines.^9^ These methods provide students with dynamic discussions that go beyond predefined case scenarios and cultivates students' analytical and active thinking. In addition, the use of online platforms can facilitate simulation‐based learning activities by reducing geographical and organisational barriers that allow for structured and synchronous activities.^10^, ^11^ Therefore, incorporating aspects of simulation can enhance students' performance and outcomes when using CBL.

When CBL is thoughtfully designed, such approaches may allow students to engage with the complexity of real‐world practice while remaining sensitive to the resource realities of their training contexts. Rather than importing pedagogical models developed elsewhere, educators in LMIC and resource‐constrained settings have an opportunity to reimagine CBL in ways that are locally grounded, culturally responsive and system‐aware. This commentary contributes to a more nuanced understanding of CBL and offers guidance for adapting educational innovations across diverse global settings. In doing so, context becomes not a limitation, but rather a catalyst for innovation.

## AUTHOR CONTRIBUTION

**Skye Nandi Adams:** Conceptualisation; writing—original draft; writing—review and editing.

## CONFLICT OF INTEREST STATEMENT

The author has no conflicts of interest to declare.

## Data Availability

Data sharing is not applicable to this article as no datasets were generated or analysed during the current study.
